# Prevalence and factors associated with insomnia among firefighting personnel in Dhaka division, Bangladesh

**DOI:** 10.1186/s12889-025-23919-2

**Published:** 2025-08-06

**Authors:** Mohammad Delwer Hossain Hawlader, Koustuv Dalal, Farah Sabrina, Md. Farhan Ibne Faruq, Nurjahan Binte Munaf, Ahmed Hossain, Md. Golam Kibria

**Affiliations:** 1 Department of Public Health, North South University, Dhaka, 1229 Bangladesh; 2 NSU Global Health Institute (NGHI), North South University, Dhaka, 1229 Bangladesh; 3 School of Health Sciences, Division of Public Health Science, Mid Sweden University, 851 70 Sundsvall, Sweden; 4 Public Health Promotion and Development Society (PPDS), Dhaka, 1205 Bangladesh; 5 College of Health Sciences, University of Sharjah, Sharjah, United Arab Emirates; 6 Centre for Development Action (CDA), Dhaka, 1207 Bangladesh

**Keywords:** Fire safety department, Firefighters, Insomnia, Insomnia severity index, Bangladesh

## Abstract

**Background:**

Firefighting is a challenging and stressful job, and firefighters face many adverse conditions while performing their duties. The study aimed to assess the prevalence of insomnia among firefighting staff working in the Dhaka division of Bangladesh and identify the factors contributing to the severity of insomnia.

**Methods:**

A cross-sectional study was conducted among a total of 406 employees of the Department of Fire Service & Civil Defense (FSCD) working in randomly selected nine districts of the Dhaka division using a simple random sampling (SRS) technique. Data were collected from the firefighting staff through face-to-face interviews. The severity of insomnia was assessed during the past 2 weeks using the Bangla version of the Insomnia Severity Index (ISI). Multivariable ordinal logistic regression (OLR) was used to identify the factors associated with insomnia among the fire service staff. All statistical analyses were performed using Stata version 17.

**Results:**

Among the 406 participants, nearly one-fourth (22.9%) suffered from moderate to severe insomnia. The results of the multivariable regression analyses showed that the firefighting staff aged 30 to 45 years (adjusted odds ratio, AOR: 2.0; 95% CI: 1.075 to 3.663) and above 45 years (AOR: 4.3, 95% CI: 1.386 to 13.039)had higher odds of insomnia than those aged below 30 years. The participants who conducted over 1,000 rescue operations had higher odds of experiencing insomnia compared to their colleagues who conducted fewer than 500 rescue operations (AOR: 2.6, 95% CI: 1.451 to 4.529). The firefighting staff with severe (AOR: 2.5, 95% CI: 1.325 to 4.551) and potentially dangerous (AOR: 3.9, 95% CI: 1.928 to 8.012) levels of workplace stress had two 2times higher odds of suffering from insomnia compared to those with minimal/mild levels of workplace stress. Furthermore, those who reported moderate (AOR: 2.0, 95% CI: 1.314 to 3.083) and severe (AOR: 2.6, 95% CI: 1.558 to 4.506) levels of PTSD were more likely to suffer from insomnia than their counterparts who reported minimal/mild levels of PTSD.

**Conclusions:**

The present study revealed that nearly one-fourth of firefighting staff working in the Dhaka division experienced moderate to severe insomnia. Several factors, including age, the number of rescue operations, workplace stress, PTSD, and chronic diseases. The findings of this study highlight the need for sleep health promotion programs in firefighting staff.

## Introduction

Professional firefighting is a distinctive and challenging occupation. Firefighting personnel face stressful, tough, and challenging working conditions and must maintain 24-hour readiness to respond immediately to emergency alarms, ensuring preparedness for fire and rescue operations [1–3]. During their working shifts, which often include night work, rotating schedules, and extended hours, firefighters are expected to remain on call and react promptly to emergency alarms [4]. The nature of firefighting, which involves prolonged shifts and exposure to high-stress environments, frequently disrupts natural sleep patterns [5].

The prevalence of sleep disturbances in firefighting personnel has been reported to range from 13.7 to 73%, depending on the different tools that were used in that specific study [6]. Emergency alarms and loud sirens often trigger the sympathetic nervous system, which may increase stress and cause sleep deprivation. Insufficient sleep is a common issue, which increases the likelihood of daytime sleepiness, fatigue, and cognitive impairments. Chronic sleep restriction can intensify existing mental health conditions, such as depression and anxiety, and contribute to insomnia [7, 8]. Firefighters often face disrupted sleep due to shift work, nocturnal calls, and sleep restriction, which interfere with their natural circadian rhythm and can lead to fatigue, cognitive impairments, and an increased risk of health issues, such as cardiovascular and metabolic disorders [1, 9, 10].

The internal circadian rhythm is regulated primarily by light exposure, but it also responds to external environmental signals, such as physical activity and melatonin production [11]. Circadian rhythm disturbances can occur when the circadian cycle fails to align with the 24-hour external environment. This imbalance can cause many health issues, such as metabolic, cognitive, cardiovascular, and gastrointestinal problems [12]. Recent research shows that 28% of the firefighters in a cohort study tested positive for obstructive sleep apnea (OSA), and 6% had a high risk of insomnia [13]. Recent research on sleep problems among US firefighters found that 59% had a sleep disturbance [1]. In Brazil, a study found that sleep disorders affected 51% of firefighters [14]. In another study, 69.9% of Iranian and Chinese firefighters had a sleep issue [15].

According to a previous study, insomnia has been linked to some other factors, including gender, educational attainment or socioeconomic class, marital status, alcohol and tobacco use, caffeine intake, and mental comorbidities like anxiety and depression [16–19]. Occupational physical activity refers to job demands, including metabolic loads and emotional burdens, in addition to mechanical loads such as prolonged workdays [20, 21]. Physical exercise at work is closely linked to unfavorable health outcomes [22] and is associated with a high prevalence of insomnia [23]. Previous research indicates that physical disease, depression, anxiety, job stress, physical activity level, and drinking status are all related to firefighters’ sleep quality [24–28].

Firefighting in Bangladesh is a high-risk profession. In recent years, Bangladesh has experienced a surge in industrial fires, primarily caused by hazardous chemical mishandling, electrical short circuits, and gas cylinder explosions. These incidents have resulted in significant fatalities and injuries, affecting both civilians and firefighters. Firefighters in Bangladesh typically work 24-hour shifts, during which they face numerous challenges. However, the impact of these demanding schedules on their sleep quality and patterns remains in obscurity. This study aimed to assess the prevalence of insomnia among firefighters who worked in the Dhaka division and identify the factors contributing to the severity of insomnia. Gaining insights into these issues would help develop strategies to address sleep-related challenges, ultimately improving the overall health, well-being, and performance of firefighting personnel in Bangladesh.

### Study design and participants

We conducted a cross-sectional study among firefighting staff who were currently employed in the Department of Fire Service & Civil Defense (FSCD) in Bangladesh. Those who worked in the field of fire service for more than a year and actively participated in fire incidents were included. On the other hand, firefighting staff who retired and had a diagnosis of significant depression illness that required medications were excluded from the study.

### Sampling and study setting

Probability sampling was used to calculate the sample size of this study. The minimum required.

The sample size for this study was calculated using a single population proportion formula,

n = $$\:\frac{{\text{z}}^{2}\text{p}\text{q}}{{\text{e}}^{2}}$$ [29, 30].

where n = desired sample size, z = standard normal deviate = 1.96 at 95% confidence interval, p = prevalence of insomnia among firefighting staff in Dhaka division (unknown) = 50.0%, and e = margin of error = 5%.

The initial sample size was 384. Considering a non-response rate of 10%, the calculated sample size for this study was 427. Although we approached 427 fire service employees for interviews, nine of them refused to participate in the study. Furthermore, 12 interviews were excluded from the 418 conducted due to incomplete information, and we ultimately analysed the data from 406 participants. Bangladesh is divided into eight administrative areas called divisions, and these divisions are further divided into 64 districts. Due to resource constraints, we conveniently selected one division, namely Dhaka division. The Dhaka division alone has 13 districts, and nine of them were randomly selected for this study: Dhaka, Narayanganj, Narshingdi, Tangail, Gazipur, Manikganj, Rajbari, Faridpur, and Munshiganj. The headquarters of the Bangladesh Fire Service and Civil Defence (FSCD) is located in the Dhaka district. The headquarters has a much larger number of employees compared to the other selected districts. Taking this fact into consideration, we allocated one-third (33.0%) of the total sample to the Dhaka district and distributed the remaining two-thirds among the eight districts equally. Out of the 406 participants, 134 were recruited from Dhaka district, and 34 were recruited from each of the eight districts. However, we compiled a list of firefighting staff from the selected nine districts at the FSCD headquarters and recruited study participants from this list using a simple random sampling (SRS) technique.

### Measures

#### Outcome variable

We assessed the severity of insomnia during the past 2 weeks using the Bangla version of the Insomnia Severity Index (ISI) [31]. The ISI is a 7-item self-report questionnaire, and each item is rated on a 5-point Likert scale (0 = no symptom, 1 = mild symptom, 2 = moderate symptom, 3 = severe symptom, and 4 = very severe symptom). The total scores range from 0 to 28, with the higher score indicating the greater severity of insomnia. The total scores were interpreted as follows: no insomnia (0 to 7), sub-threshold insomnia (8 to 14), moderate insomnia (15 to 21), and severe insomnia (22 to 28) [32]. The ISI was found to have good reliability (Cronbach’s alpha = 0.891 and mean inter-item correlation = 0.539).

#### Sociodemographic variables

In this study, sociodemographic factors included age (< 30 years, 30 to 45 years, or > 45 years), marital status (married or unmarried), education level (secondary, higher secondary, or bachelor’s degree and above), household size, monthly household income (≤ 30,000 Bangladeshi taka BDT, 30,001 to 40,000 BDT, or > 40,000 BDT), and smoking status (smoker or non-smoker).

#### Occupational variables

Occupational variables included designation (managerial staff, firefighter, driver), job tenure (< 5 years, 5 to 10 years, or > 10 years), and no. of fire rescue operations (< 500, 500 to 1,000, or > 1,000), and levels of workplace stress (relatively calm, fairly low, moderate, severe, or potentially dangerous). The workplace stress was evaluated using the Workplace Stress Scale (WSS) [33]. The WSS consists of eight items, each rated on a five-point Likert scale: 1 = never, 2 = rarely, 3 = sometimes, 4 = often, and 5 = very often. Item numbers 6, 7, and 8 are reverse-scored, and the total score ranges between 8 and 40. The total score of the scale was categorized into five levels: relatively calm (15 or lower), fairly low (16 to 20), moderate (21 to 25), severe (26 to 30), and potentially dangerous (31 to 40). For the convenience of interpreting the results, we converted these five categories into four: relatively calm/fairly low, moderate, severe, and potentially dangerous.

#### Health-related variables

In this study, chronic disease status (present or absent) and levels of post-traumatic stress disorder (PTSD) (no, mild, moderate or severe) were considered as health-related variables. The participants were asked whether they had diabetes mellitus, hypertension, chronic heart disease, chronic kidney disease, asthma, pneumonia, chronic obstructive pulmonary disease (COPD), and tuberculosis. Those who reported having at least one of these diseases were categorized as having chronic diseases. The symptoms of PTSD among the firefighting personnel were assessed using the Short PTSD Rating Interview (SPRINT) scale [34]. The SPRINT is an 8-item self-report measure for screening PTSD and other aspects, such as somatic concerns, stress vulnerability, and functional impairment. Each item is rated on a 5-point Likert scale (0 = not at all, 1 = a little bit, 2 = moderately, 3 = quite a lot, and 4 = very much), with a total score ranging from 0 to 32. The levels of PTSD were categorized as minimal (6 or lower), mild (7 to 10), moderate (11 to 17), and severe (18 to 32) [35]. These four categories were further combined into three categories: minimal/mild, moderate, and severe for easier interpretations of the results.

### Data collection

Data were collected from the firefighting staff through face-to-face interviews, following some inclusion and exclusion criteria. Those who have worked in this field for over a year and actively participated in fire incidents were included. However, firefighting staff who were retired or declined to take part in the study and had a diagnosis of significant depression illness that required medication were excluded from the study. A pre-tested questionnaire was used to gather data for this study to determine the prevalence and factors associated with job stress among firefighting staff in Bangladesh. The questionnaires were first developed in English and then translated into Bangla. Finally, a third party translated it back to ensure the original translation was accurate. The study was carried out by the Institutional Research Ethics and the Declaration of Helsinki, and its latest amendment was in October 19, 2024, which is comparable to its ethical standards. The Institutional Review Board (IRB) of North South University has approved this study (2023/OR-NSU/IRB/1228). Written informed consent was obtained before each interview, and the participants could withdraw from the study at any time.

### Statistical analysis

All statistical analyses were performed using Stata version 17. Frequencies and percentages were used to summarize the study sample’s sociodemographic, occupational, and health-related characteristics. The bivariate associations between insomnia and categorical variables were determined using the chi-square test of independence and Fisher’s exact test. Fisher’s exact test was applicable for those categorical variables with an expected frequency of less than five observations in a cell. In this study, insomnia was the outcome variable and was measured using an ordinal scale with four categories: no, sub-threshold, moderate, and severe. Considering the ordinal data of the outcome variable, we performed multivariable ordinal logistic regression (OLR) to identify the factors associated with insomnia among the firefighting staff. We included all the independent variables in the OLR model and performed the regression analysis using the backward selection procedure. Before running the analysis, we evaluated the model’s fitness using the ordinal Hosmer-Lemeshow (HL) test [36]. The p-value for the HL test was 0.873, which is greater than 0.05, indicating that the model was a good fit for the data [37]. However, the basic assumption of an OLR model is the proportional odds assumption, which means that the effects of all independent variables are constant across all categories of the outcome variable. The Brant test was used to assess the proportional odds assumption (POA), and the test results indicated that the assumption was satisfied at a p-value greater than 0.05 [38]. Multicollinearity was tested using the variance inflation factor (VIF), and we observed a VIF of less than 5 in each independent variable with a mean VIF of 1.91, indicating that there was no significant multicollinearity between the independent variables [39]. The regression analysis results were presented as adjusted odds ratios (AORs) and 95% confidence intervals (CIs). All statistics were tested using a two-sided test, and a p-value of < 0.05 was considered statistically significant.

## Results

Table 1 depicts the sociodemographic, occupational, and health-related characteristics of study participants. A total of 406 fire service workers participated in this study, of whom the majority (55.9%) were below 30 years of age. As regards the educational level, more than half of the participants (58.9%) were firefighters who had completed higher secondary education. About three-fourths of the participants (74.9%) were married. The majority of the fire service workers claimed that their household size was 4 to 6 persons (67.2%), and their monthly household income was ≤ 30,000 BDT (52.2%). Among the participants, (73.2% reported themselves as non-smokers). Interestingly, an overwhelming majority of the participants (80.8%) were firefighters by designation. The largest portion of the participants (35.2%) worked in the fire service sector for more than 10 years, followed by 5 to 10 years of tenure (34.0%). In terms of the number of fire rescue operations, 40.1% of the participants participated in more than 500 rescue operations. It is alarming that more than one-third of the firefighting staff (37.2%) reported severe to potentially dangerous levels of workplace stress. Among the firefighters, 33.3% had a history of chronic diseases, and 18.2% suffered from severe levels of PTSD.

**Table 1:** Sociodemographic, occupational, and health-related characteristics of study participants

Variable	Category	Frequency	Percentage
Age group	< 30 years	227	55.9
	30 to 45 years	164	40.4
	> 45 years	15	3.7
Education level	Secondary	99	24.4
	Higher Secondary	243	59.9
	Bachelor’s degree and above	64	15.8
Marital status	Married	304	74.9
	Unmarried	102	25.1
Household size	1 to 3 persons	39	9.6
	4 to 6 persons	273	67.2
	≥ 7 persons	94	23.2
Monthly household income	≤ 30,000 BDT	212	52.2
	31,0001 to 50,000 BDT	149	36.7
	> 50,000 BDT	45	11.1
Smoking status	Smoker	109	26.8
	Non-smoker	297	73.2
Designation	Firefighter	328	80.8
	^a^Managerial staff	37	9.1
	Driver	36	8.9
	Others	5	1.2
Job tenure	< 5 years	125	30.8
	5 to 10 years	138	34.0
	> 10 years	143	35.2
No. of rescue operations	< 500	163	40.1
	500 to 1,000	115	28.3
	> 1,000	128	31.5
Levels of workplace stress	Relatively calm/Fairly low	70	17.2
	Moderate	185	45.6
	Severe	99	24.4
	Potentially dangerous	52	12.8
Chronic disease	Present	135	33.3
	Absent	271	66.7
Levels of PTSD	Minimal/Mild	180	44.3
	Moderate	151	37.2
	Severe	75	18.5

^a^Managerial staff includes the assistant director, senior station officer, station officer, warehouse inspector, and sub-officer

**Fig. 1:**
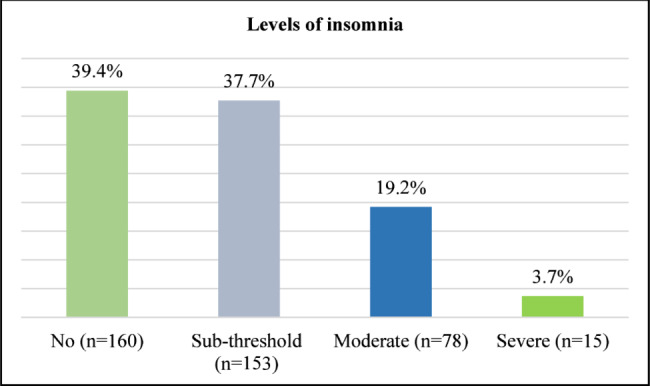
Levels of insomnia among firefighting personnel

Figure 1 presents the levels of insomnia among the firefighting personnel studied. Among the 406 participants, 22.9% suffered from moderate to severe insomnia and 37.7% experienced sub-threshold insomnia. On the other hand, about two-fifths (39.4%) of the respondents reported no insomnia.

Table 2 presents the bivariate associations of insomnia with sociodemographic, occupational, and health-related characteristics among the firefighting staff. The prevalence of moderate to severe insomnia was higher among the participants aged above 45 years (53.3%), and there was a significant association between the levels of insomnia and the age group (*p* < 0.001). Among the firefighting staff, those who conducted more than 1,000 rescue operations had a higher prevalence of moderate to severe insomnia (29.7%). A significant association was observed between the levels of insomnia and the number of rescue operations (*p* = 0.015). Moderate to severe insomnia was found to be higher among those who experienced potentially dangerous levels of workplace stress (32.7%). The levels of insomnia were significantly associated with the levels of workplace stress (*p* = 0.004). More than one-third of the participants with a history of chronic diseases (37.3%) suffered from moderate to severe insomnia, and there was a statistically significant association between the levels of insomnia and chronic disease status (*p* = 0.005). Moreover, the participants who reported severe levels of PTSD symptoms had a higher prevalence of moderate to severe insomnia(34.7%). It was also observed that levels of insomnia were significantly associated with the levels of PTSD (*p* < 0.001).

**Table 2:** Bivariate associations of insomnia with sociodemographic, occupational, health-related characteristics among firefighting personnel

Variable	Levels of insomnia				^‡^p-value
	No	Sub-threshold	Moderate	Severe	
Age group	n (%)	n (%)	n (%)	n (%)	
< 30 years	103 (45.4)	84 (37.0)	35 (15.4)	5 (2.2)	**< 0.001**
30 to 45 years	51 (31.1)	68 (41.5)	35 (21.3)	10 (6.1)	
> 45 years	6 (40.0)	1 (6.7)	8 (53.3)	0 (0.0)	
Education level					
Secondary	36 (36.4)	39 (39.4)	18 (18.2)	6 (6.1)	0.686
Higher Secondary	102 (42.0)	89 (36.6)	45 (18.5)	7 (2.9)	
Bachelor’s degree or above	22 (34.4)	25 (39.1)	15 (23.4)	2 (3.1)	
Marital status					
Married	121 (39.8)	111 (36.5)	60 (19.7)	12 (3.9)	0.874
Unmarried	39 (38.2)	42 (41.2)	18 (17.6)	3 (2.9)	
Household size					
1 to 3 persons	15 (38.5)	18 (46.2)	5 (12.8)	1 (2.6)	0.723
4 to 6 persons	113 (41.4)	96 (35.2)	53 (19.4)	11 (4.0)	
≥ 7 persons	32 (34.0)	39 (41.5)	20 (21.3)	3 (3.2)	
Monthly household income					
≤ 30,000 BDT	81 (38.2)	81 (38.2)	42 (19.8)	8 (3.8)	0.869
30,0001 to 50,000 BDT	57 (38.3)	59 (39.6)	27 (18.1)	6 (4.0)	
> 50,000 BDT	22 (48.9)	13 (28.9)	9 (20.0)	1 (2.2)	
Smoking status					
Smoker	37 (33.9)	50 (45.9)	19 (17.4)	3 (2.8)	0.239
Non-smoker	123 (41.4)	103 (34.7)	59 (19.9)	12 (4.0)	
Designation					
Firefighter	128 (39.0)	124 (37.8)	66 (20.1)	10 (3.0	0.505
^a^Managerial staff	17 (45.9)	14 (37.8)	4 (10.8)	2 (5.4)	
Driver	13 (36.1)	13 (36.1)	8 (22.2)	2 (5.6)	
Others	2 (40.0)	2 (40.0)	0 (0.0)	1 (20.0)	
Job tenure					
< 5 years	56 (44.8)	47 (37.8)	18 (14.4)	4 (3.2)	0.285
5 to 10 years	58 (42.0)	49 (35.5)	25 (18.1)	6 (4.3)	
> 10 years	46 (32.2)	57 (39.9)	35 (24.5)	5 (3.5)	
No. of rescue operations					
< 500	80 (49.1)	58 (35.6)	20 (12.3)	5 (3.1)	**0.015**
500 to 1,000	40 (34.8)	45 (39.1)	27 (23.5)	3 (2.6)	
> 1,000	40 (31.3)	50 (39.1)	31 (24.2)	7 (5.5)	
Levels of workplace stress					
Relatively clam/ Fairly low	35 (50.0)	27 (38.6)	6 (8.6)	2 (2.9)	**0.004**
Moderate	81 (43.8)	62 (33.5)	37 (20.0)	5 (2.7)	
Severe	34 (34.3)	39 (39.4)	23 (23.2)	3 (3.0)	
Potentially dangerous	10 (19.2)	25 (48.1)	12 (23.1)	5 (9.6)	
Chronic disease status					
Present	16 (27.1)	21 (35.6)	16 (27.1)	6 (10.2)	**0.005** ^†^
Absent	144 (41.5)	132 (28.0)	62 (17.9)	9 (2.6)	
Levels of PTSD					
Minimal/Mild	100 (55.6)	49 (27.2)	23 (12.8)	8 (4.4)	**< 0.001**
Moderate	45 (29.8)	70 (46.4)	32 (21.2)	4 (2.6)	
Severe	15 (20.0)	34 (45.3)	23 (30.7)	3 (4.0)	

^a^Managerial staff includes the assistant director, senior station officer, station officer, warehouse inspector, and sub-officer; ^‡^Fisher’s exact test; ^†^Pearson’s chi-square test; Bold values indicate significant results

Table 3 presents the results of multivariable ordinal logistic regression analysis to identify the factors associated with insomnia among the firefighting staff. The participants aged 30 to 45 years and above 45 years had 2.0 (95% CI: 1.075 to 3.663) and 4.3 (95% CI: 1.386 to 13.039) times higher odds to experience insomnia, respectively compared to those aged below 30 years. The firefighting staff who conducted more than 1,000 rescue operations were at 2.6 times higher odds of having insomnia compared to their colleagues who conducted less than 500 rescue operations (95% CI: 1.451 to 4.529). The firefighting staff who reported severe and potentially dangerous levels of workplace stress had 2.5 (95% CI: 1.325 to 4.551) and 3.9 (95% CI: 1.928 to 8.012) times higher odds of experience insomnia compared to their counterparts who reported relatively calm/fairly low levels of workplace stress). Similarly, the participants with moderate and severe levels of PTSD had 2.0 (95% CI: 1.314 to 3.083) and 2.6 (95% CI: 1.558 to 4.506) times higher odds of suffering from insomnia, respectively than those with minimal/mild levels of insomnia.

**Table 3:** Ordinal logistic regression analysis of factors associated with insomnia among firefighting personnel

Variable	AOR	95% CI	*p*-value
Age group			
< 30 years	Reference		
30 to 45 years	2.0	1.075 to 3.663	0.029*
> 45 years	4.3	1.386 to 13.039	0.011*
No. of rescue operations			
< 500	Reference		
500 to 1,000	1.5	0.943 to 2.548	0.084
> 1,000	2.6	1.451 to 4.529	0.001**
Levels of workplace stress			
Minimal/Mild	Reference		
Moderate	1.6	0.929 to 2.779	0.090
Severe	2.5	1.325 to 4.551	0.004**
Potentially dangerous	3.9	1.928 to 8.012	< 0.001***
Levels of PTSD			
Minimal/Mild	Reference		
Moderate	2.0	1.314 to 3.083	0.001**
Severe	2.6	1.558 to 4.506	< 0.001***

AOR, Adjusted Odds Ratio; CI, Confidence Interval; *p < 0.05; **p < 0.01; *p* < 0.001.

## Discussion

Insomnia is a significant concern among firefighting personnel, given the demanding nature of their work and the psychological stressors they face. The findings of the present study identified four factors associated with insomnia in this unique population: age, number of rescue operations, workplace stress, and post-traumatic stress disorder (PTSD).

In this study, we investigated the levels of insomnia among firefighting personnel in Bangladesh. Of the 406 study participants, 22.9% had moderate to severe insomnia. In addition, the participants reported having below-adequate sleep quality; the prevalence of poor sleep in a study was 69.9%, consistent with that found among Chinese and Iranian firefighting staff [15]. In the U.S. firefighting staff, the prevalence of insomnia was around 59% [1]. In Canada, a recent study indicated that 69.2% of participants reported having below-adequate sleep quality or insomnia. In Canada, the same research also reported that 21% of fighting personnel screened positive for clinical insomnia [7]. In another study, the prevalence of insomnia in South Korea reported was 48.7% [40]. In Brazil, a study conducted in 2012 reported that 51% of firefighters were suffering from a sleep disorder [41].

In our study, we found a positive association between age and insomnia. As age increases, the likelihood of experiencing insomnia also rises. from our study, we found that the risk of insomnia rises significantly with age. Participants those aged over 45 years had a 4.3-fold increase in the odds of having insomnia. These findings suggest a strong relationship between increasing age and the odds of developing insomnia. The results reinforce that older age groups, particularly those over 45, are at substantially higher risk of insomnia among firefighting staff. This trend may reflect age-related physiological and psychological changes that disrupt sleep, such as increased stress, comorbidities, or changes in circadian rhythm. similarly, in a large-scale study involving 9,788 Korean firefighters, 9.1% were found to suffer from insomnia. Older adults (≥ 50 years) were most affected, with more than 1 in 10 reporting insomnia, compared to only 7% among those aged 20–29. The statistically significant trend (*p* < 0.001) suggests that age is a significant risk factor for insomnia in this population [42]. Our study found that firefighting staff who conducted more than 1,000 rescue operations were more likely to experience insomnia compared to their colleagues who conducted. A study conducted in Korea indicated that the likelihood of poor sleep decreased significantly with longer years of service. It was found in the study that 52.5% of firefighters with less than 10 years of service reported poor sleep, compared to 51.3% with 10 to 20 years of service, and 37.7% with over 20 years of service. These findings highlight the impact of both the intensity of rescue operations and years of service on sleep disorders among male firefighters [25]. Furthermore, another study in Taiwan revealed that firefighters who worked for more than 5 years were 2.5 times more likely to experience poor sleep quality or insomnia than those who worked for five years or less. This association is statistically significant, which indicates a strong and reliable relationship between years of service and sleep disturbances [43]. To mitigate sleep disturbances among firefighters, fire departments should regularly monitor individual workloads and implement strategies that promote adequate rest and proper sleeping time, thereby enhancing overall health and better job performance. The present study found that firefighting staff who had moderate to severe post-traumatic stress disorder (PTSD) had higher odds of experiencing insomnia compared to those who had minimal/mild levels of PTSD. A recent study carried out in Thailand showed that the prevalence of PTSD among firefighters was 6.4%, and more than 80% of them had poor sleep quality [44]. The same study revealed that PTSD status constitutes an increased risk of poor sleep quality among urban firefighters. At the same time, a Canadian study explored insomnia as a risk factor for PTSD, showing that firefighters who screened positive for clinical insomnia had 4.98 to 8.53 times higher odds of screening positive for PTSD [7]. Regular mental health screenings for firefighters are essential to detect early signs of PTSD and sleep disturbances, facilitating timely interventions that can reduce the intensity of insomnia among firefighting staff.

According to the findings of the present study, the firefighting staff who reported severe to potentially dangerous levels of workplace stress were at greater risk of experiencing insomnia compared to their counterparts who reported relatively calm/fairly low levels of workplace stress. A study of 705 male firefighters in Korea found that those with the highest tertile reported work-related stress experienced the most significant sleep dysfunction, which is consistent with our findings. Another study conducted among 154 career firefighters in Northern California showed that firefighters who reported the highest job stress scores were 3.7 times more likely to experience sleep disturbances compared to those who reported the lowest job stress scores. Firefighters encounter unique occupational stressors that can adversely affect their sleep quality and overall well-being. Implementing a combination of individual practices and organizational strategies is crucial to fostering a supportive environment that prioritizes their health and resilience.

### Strengths and limitations

One main strength of this study is the use of a validated Bangla version of the Insomnia Severity Index (ISI). This allowed the authors to reliably assess the severity of insomnia in a Bangladeshi population, which is crucial for understanding and addressing sleep problems in this specific demographic. Another strength of the current study is that this cross-sectional study is the first, to our knowledge, which investigated insomnia in Bangladeshi firefighting workforce. From the perspective of occupational health policies, it is crucial to understand the severity of insomnia in an occupational group like firefighting workforce. This study also has several limitations. First, the study was conducted in one division in Bangladesh. So, the inclusion of firefighting staff from other divisions of the country could lead to a wider range of perspectives and experiences. Second, clinical assessment was not conducted to diagnose insomnia, workplace stress, or PTSD. Third, the cross-sectional design of this study design cannot establish causal inferences. Therefore, longitudinal studies can be conducted in the future to provide deeper insights into the dynamics of mental health among firefighting personnel in Bangladesh. Finally, some data of the current study relied on past events and experiences, which could lead to recall bias.

## Conclusions

This study found that nearly one-fourth of firefighting personnel working in the Dhaka division suffered from insomnia, with risk factors including age, number of rescue operations, workplace stress, and PTSD. The findings align with international research, underscoring the need for targeted interventions such as wellness programs and stress management. To enhance sleep quality and mental health, the firefighting community could benefit from workplace wellness programs, stress management interventions, and routine screening for insomnia and psychological conditions. These measures are essential for safeguarding the well-being and operational efficiency of firefighting personnel. Future longitudinal studies with clinical assessments are recommended to deepen understanding and improve firefighter well-being.

## Data Availability

The data underlying the results presented in this study will be provided at a reasonable request by Dr. Mohammad Delwer Hossain Hawlader. Email: mohammad.hawlader@northsouth.edu.
